# A toolkit for covalent docking with GOLD: from automated ligand preparation with KNIME to bound protein–ligand complexes

**DOI:** 10.1093/bioadv/vbac090

**Published:** 2022-11-29

**Authors:** Laurianne David, Anissa Mdahoma, Natesh Singh, Sébastien Buchoux, Emilie Pihan, Constantino Diaz, Obdulia Rabal

**Affiliations:** Evotec SE, Molecular Architects, Integrated Drug Discovery, Toulouse 31036, France; Evotec SE, Molecular Architects, Integrated Drug Discovery, Toulouse 31036, France; Evotec SE, Molecular Architects, Integrated Drug Discovery, Toulouse 31036, France; Evotec SE, Scientific Data Management, Toulouse 31036, France; Evotec SE, Molecular Architects, Integrated Drug Discovery, Toulouse 31036, France; Evotec SE, Molecular Architects, Integrated Drug Discovery, Toulouse 31036, France; Evotec SE, Molecular Architects, Integrated Drug Discovery, Toulouse 31036, France; Pharmacelera, Torre R, 4a Planta, Despatx A05, Parc Científic de Barcelona (PCB), Barcelona 08028, Spain

## Abstract

**Motivation:**

Current covalent docking tools have limitations that make them difficult to use for performing large-scale structure-based covalent virtual screening (VS). They require time-consuming tasks for the preparation of proteins and compounds (standardization, filtering according to the type of warheads), as well as for setting up covalent reactions. We have developed a toolkit to help accelerate drug discovery projects in the phases of hit identification by VS of ultra-large covalent libraries and hit expansion by exploration of the binding of known covalent compounds. With this application note, we offer the community a toolkit for performing automated covalent docking in a fast and efficient way.

**Results:**

The toolkit comprises a KNIME workflow for ligand preparation and a Python program to perform the covalent docking of ligands with the GOLD docking engine running in a parallelized fashion.

**Availability and implementation:**

The KNIME workflow entitled ‘*Evotec_Covalent_Processing_forGOLD.knwf*’ for the preparation of the ligands is available in the KNIME Hub https://hub.knime.com/emilie_pihan/spaces.

**Supplementary information:**

Supplementary data are available at *Bioinformatics Advances* online.

## 1 Introduction

Covalent inhibition of disease-related target proteins has acquired increasing attention in the last two decades (Singh *et al.*, 2011). Covalent inhibitors are typically characterized by the formation of a chemical bond between an electrophilic moiety (warhead) on the ligand part and a nucleophilic residue, most commonly cysteine, of the protein target. The formation of the bond between protein and ligand can be either reversible or irreversible. Covalent inhibitors have many advantages as drug candidates: a prolonged duration of action, improved potency (Bradshaw *et al.*, 2015), and exquisite selectivity when nonconserved protein nucleophiles are targeted (Cohen *et al.*, 2005). There are three main approaches for covalent ligand discovery: (i) high-throughput screening, in which small molecules that react covalently with a target are identified by screening of existing libraries. However, it is challenging to identify selective reactive hits for a target among the unselective hits (Baell and Holloway, 2010; Kathman *et al.*, 2014; Potashman and Duggan, 2009); (ii) fragment-based screening, in which low molecular weight chemical probes are identified. A major limitation of this approach can be the weak binding affinity of the electrophilic fragments; (iii) structure-based virtual screening (SBVS) (Muller *et al.*, 2022), in which large virtual compound libraries are computationally screened to predict molecules that bind favorably to the target. SBVS is widely used for the discovery of both covalent and noncovalent ligands (Glick and Jacoby, 2011; Totrov and Abagyan, 2008).

With the growing interest in covalent drug discovery, there is an urgent need for a platform that could efficiently support the SBVS of covalent compounds in an automated way. This is not offered by current covalent docking tools, e.g. CovDock, GOLD, DOCKTITE, AutoDock4. For example, the execution of covalent docking requires a manual description of the reactive atoms and reaction type, as well as manual preparation of ligand and protein structure files. This makes these common software packages difficult to use for large-scale covalent SBVS. We report the development of a toolkit that incorporates the commonly used GOLD docking software that was created to address the current limitations of covalent SBVS. It consists of a KNIME workflow for ligand library preparation (screenshot on Supplementary Appendix S1) and a Python program that performs parallel covalent docking of ligands across CPUs using GOLD.

We have also shared test cases that show our toolkit is straightforward to use and efficient to run and can be successfully applied in covalent SBVS campaigns with minimal human attention.

## 2 Implementation

**Fig. 1.**
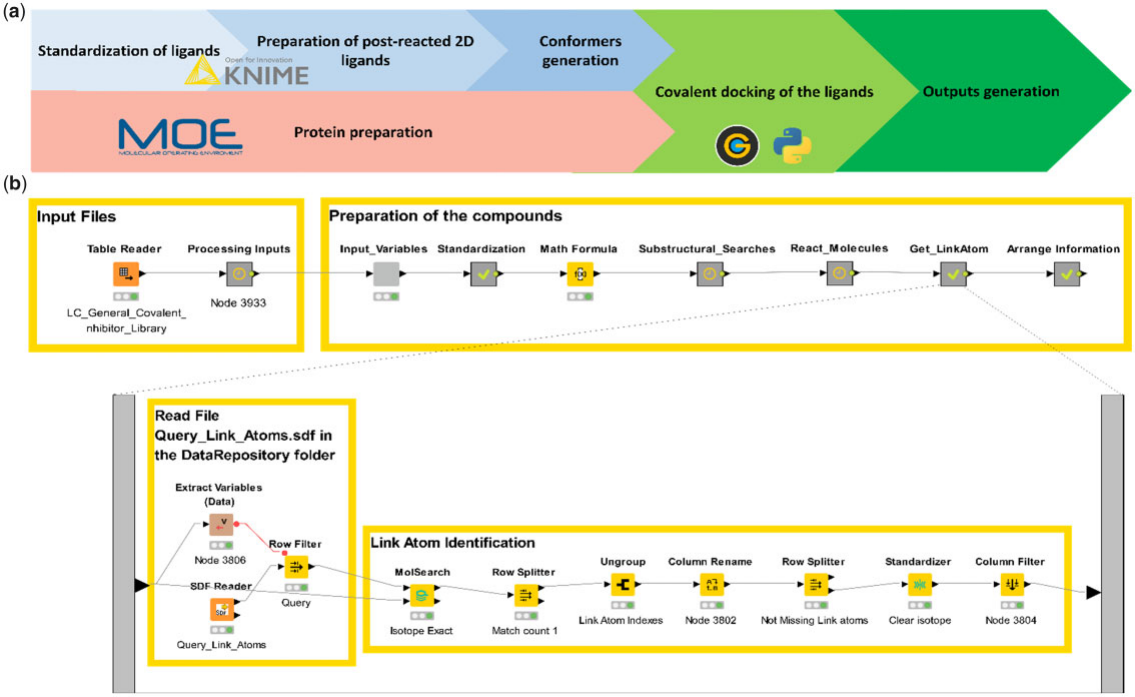
(**a**) Workflow describing the developed automated covalent docking protocol. The main steps are the following: (i) preparation of the ligands, (ii) preparation of the protein system, (iii) covalent docking of the ligands and (iv) output generation. (**b**) Knime snapshot of the main workflow of the covalent docking, with a focus on the Get_LinkAtom metanode, in which the link atom is automatically identified

To achieve covalent docking with GOLD, three object types are needed: (i) compounds must be provided as SD file(s), these are then processed in the KNIME workflow as described below, (ii) a protein system must be prepared as a .mol2 file and (iii) the covalent docking of the compounds is performed using a Python script which uses GOLD and provides an SD file as an output. The workflow describing the automated covalent docking protocol is shown in Figure 1.

### 2.1 Preparation of the compounds

#### 2.1.1 Standardization of compounds

Compounds must be provided as an SD file to the KNIME workflow. Standardization of compounds is performed using *ChemAxon* extensions in KNIME v.4.2.4. Isotopes, radicals and molecules with nonstandard chemical elements (different from H, C, O, N, S, P, B and halogens) are discarded (*Chemical Terms* node). Missing structures are discarded by calculating the molecular weight of the compounds and filtering out null structures. If fragmented structures are found, then the largest fragment is considered, salts and solvents are then removed. Mesomers (e.g. nitro, sulfone, sulfoxide) are standardized, and compounds are neutralized, aromatized and wiggly bonds are transformed into single bonds (*Standardizer* node). Major tautomers (at pH 7.4) and tetrahedral stereoisomers (enumeration up to 1000 stereoisomers, which accounts for up to 9 undefined chiral centers) are finally generated, and the major microspecies at pH 7.4 are retained. A part of this process is presented in Supplementary Figure S1.

#### 2.1.2 Identification of warheads

To carry out the appropriate covalent reaction, one must first identify the covalent reactive group, also referred to as a warhead. A total of 68 reactive warheads, collected from the literature (Supplementary Citation S1), were identified by substructural search (*MolSearch node*). The full list of warheads is tabulated in Supplementary Appendix S2. These warheads are related to the most common residues: Cys, Lys, Ser, Tyr, Thr, Asp and Glu. Not all of the warheads are reactive for all nucleophilic residues, so the list is conveniently handled depending on the user selection of the protein residue. Compounds containing one chemical warhead were retained, while compounds bearing several warheads, the same warhead with frequency >1, or unidentified warheads were discarded.

#### 2.1.3 Reaction of the compound

Reactions files (*.rxn) were drawn for each warhead, resulting in a total of 98 reactions. Post-reacted compounds are obtained by applying the corresponding reaction on the compound (*Reactor node*) (Supplementary Fig. S2). Each reaction was defined to introduce a different link atom such as sulfur for cysteine or oxygen for threonine (Supplementary Figs S3 and S4).

#### 2.1.4 Identification of the ligand link atom

The numbers attached to the compound’s atom to be covalently bound to the protein during the docking are extracted (only records with one atom were kept). To avoid interfering groups during this step, isotopic labels of reacting atoms are introduced and cleared after link atom number retrieval.

#### 2.1.5 Output generation for docking

After all of these steps, a 2D SDF file of post-reacted compounds is automatically generated, with information on the link atom number, a unique compound identifier, a counter for the different enumerated stereoisomers, SMILES of the original compound and other relevant fields from the input SDF file (i.e. biological activity). Conformers were generated with Corina, enumerating up to two stereocenters for undefined chiral centers that might arise after the reaction.

### 2.2 Preparation of the target proteins

Any software can be used to prepare the protein system. In this work, MOE was used to generate a prepared .mol2 file. The target protein structures are prepared using two pre-processing tools with default settings: (i) *Structure Preparation* to correct structural issues such as the alternate location of the residues, and atoms with the incorrect number of hydrogens, (ii) *Protonate3D* to choose the appropriate protonation states of the residues and add hydrogens in the macromolecular structure given its 3D coordinates.

### 2.3 Covalent docking of the compounds

There are two prominent approaches for investigating the binding mechanism of covalent compounds: (i) classical (noncovalent) docking in which we exhaustively sample the ligand poses followed by filtering of the poses based on a distance cutoff (∼5 Å) between the two atoms involved in the covalent bond formation; and (ii) covalent docking, a two-step mechanism, in which the docking algorithm first generates a noncovalent complex with the ligand warhead positioned in close proximity and correct orientation to the targeted nucleophilic residue. In the second step, a covalent bond is formed according to the provided chemical reaction between the electrophilic warhead and nucleophile. The classical docking approach is computationally less expensive as compared to covalent docking (Scarpino *et al.*, 2018). Here, we used an in-house developed ‘*covalent_docking_helper.py*’ script for performing covalent docking of different datasets. This program carries out covalent docking using four different GOLD scoring functions: GoldScore, ASP, ChemScore and PLP (explanations and references are available in Supplementary Citation S2). The script’s sub-command ‘*prepare*’ generates individual .conf files for each ligand in the input .sdf file, the corresponding individual input ligand files, and outputs an execution command submitting the jobs to the cluster. Parallel docking of the compounds is executed by calling the ‘*gold_auto*’ script from GOLD. In the end, an additional function ‘*process*’ is used to generate the covalently bound protein-compound complexes from the docking poses. In addition, we evaluated the complementary approach based on the noncovalent docking of compounds on two targets OTUB2 and NUDT7. The virtual screening (VS) metrics (AUC, enrichment factors) to assess performance in calibration can be calculated in KNIME using a specific section of the previously published workflow (Pihan *et al.*, 2021).

## 3 Application to test cases

In this section, we would like to give a general overview of lessons learnt by study cases on which we applied our covalent docking tool. We would like the reader to note that our aim is not to provide an in-depth analysis of our results, but to support the correct implementation of our preparation tools. This was applied to process 9146 molecules related across six different targets. Overall, our covalent docking workflow runs correctly, and provided excellent performance with any of the scoring functions available through GOLD, with an average AUC = 0.72, the best case being an AUC = 0.98 with the ASP scoring function. The performance of classical docking on two targets showed comparable AUCs with respect to covalent docking. However, the latter yielded better enrichment factors indicating that the developed covalent docking protocol could be beneficial in the early recovery success of VS campaigns. Case studies are detailed in Supplementary Tables S1–S5 and Figures S5–S8.

Here, our aim was not to assess the performance of GOLD, but the correct functioning of our toolkit. Limitations inherent to covalent docking (e.g. protein flexibility, prediction of intrinsic reactivity of the covalent compounds) were of course observed. In conclusion, our toolkit is appropriate for covalent SBVS and can significantly improve the speed of the calculations via parallel docking. Our toolkit is currently being used for prospective covalent screening in external projects.

## Supplementary Material

vbac090_Supplementary_Data

## Data Availability

The open-source Knime was used to build the main workflow. Ligands preparation was done using ChemAxon extension through Knime and Corina. The target protein was prepared with the commercial software MOE. Docking was performed with GOLD, which is proprietary. We would like to advise the users that Knime, ChemAxon and GOLD are prerequisite for running our workflow and that the last two do require licenses. Corina and MOE can easily be replaced by open-source software (respectively, RDKit and Autodock for example). The ‘*covalent_docking_helper.py*’ script is written in Python 3.7. Python code depends on the Python Standard Library and CCDC Python API. The program can be run on Linux, MacOS and Windows operating systems. The source code is available at https://gitlab.com/seb-buch/covalent_docking_helper. The program is licensed under the GNU GPLv3. *Conflict of Interest*: none declared.

## References

[vbac090-B1] Baell J.B. , Holloway G.A. (2010) New substructure filters for removal of pan assay interference compounds (PAINS) from screening libraries and for their exclusion in bioassays. J. Med. Chem., 53, 2719–2740. 20131845 10.1021/jm901137j

[vbac090-B2] Bradshaw J.M. et al (2015) Prolonged and tunable residence time using reversible covalent kinase inhibitors. Nat. Chem. Biol., 11, 525–531. 26006010 10.1038/nchembio.1817 PMC4472506

[vbac090-B3] Cohen M.S. et al (2005) Structural bioinformatics-based design of selective, irreversible kinase inhibitors. Science, 308, 1318–1321. 15919995 10.1126/science1108367 PMC3641834

[vbac090-B4] Glick M. , Jacoby E. (2011) The role of computational methods in the identification of bioactive compounds. Curr. Opin. Chem. Biol., 15, 540–546. 21411361 10.1016/j.cbpa.2011.02.021

[vbac090-B5] Kathman S.G. et al (2014) A fragment-based method to discover irreversible covalent inhibitors of cysteine proteases. J. Med. Chem., 57, 4969–4974. 24870364 10.1021/jm500345q PMC4113264

[vbac090-B6] Muller C. et al (2022) Artificial intelligence, machine learning, and deep learning in real-life drug design cases. Methods Mol. Biol., 2390, 383–407. 34731478 10.1007/978-1-0716-1787-8_16

[vbac090-B7] Pihan E. et al (2021) Fine tuning for success in structure-based virtual screening. J. Comput. Aided Mol. Des., 35, 1195–1206. 34799816 10.1007/s10822-021-00431-4

[vbac090-B8] Potashman M.H. , Duggan M.E. (2009) Covalent modifiers: an orthogonal approach to drug design. J. Med. Chem., 52, 1231–1246. 19203292 10.1021/jm8008597

[vbac090-B9] Scarpino A. et al (2018) Comparative evaluation of covalent docking tools. J. Chem. Inf. Model., 58, 1441–1458. 29890081 10.1021/acs.jcim.8b00228

[vbac090-B10] Singh J. et al (2011) The resurgence of covalent drugs. Nat. Rev. Drug Discov., 10, 307–317. 21455239 10.1038/nrd3410

[vbac090-B11] Totrov M. , Abagyan R. (2008) Flexible ligand docking to multiple receptor conformations: a practical alternative. Curr. Opin. Struct. Biol., 18, 178–184. 18302984 10.1016/j.sbi.2008.01.004 PMC2396190

