# Supplementary material for: A toolkit for covalent docking with GOLD: from automated ligand preparation with KNIME to bound protein–ligand complexes
Source: Bioinform Adv. 2022 Nov 29;2(1):vbac090. doi: 10.1093/bioadv/vbac090 (PMC9722222; doi:10.1093/bioadv/vbac090)
Supplement: vbac090_Supplementary_Data [file vbac090_supplementary_data.zip › supporting_information.pdf]

Supporting Information

A toolkit for covalent docking with GOLD: from automated ligand preparation with KNIME to bound protein-ligand complexes.

Laurianne David[1,#], Anissa Mdahoma[1,#], Natesh Singh[1,#,*], Sébastien Buchoux[2], Emilie Pihan[1], Constantino Diaz[1], Obdulia Rabal[1,3,*]

[1]Evotec SE, Molecular Architects, Integrated Drug Discovery, Campus Curie, 195 Route d'Espagne, 31036 Toulouse, France

[2]Evotec SE, Scientific Data Management, Campus Curie, 195 Route d'Espagne, 31036 Toulouse, France

[3]Pharmacelera. Torre R, 4a planta, Despatx A05. Parc Científic de Barcelona (PCB). c/ Baldiri Reixac 4-8. 08028 Barcelona, Spain

[#]These authors contributed equally to this work.

*To whom correspondence should be addressed.

Contact : natesh.singh@evotec.com, obdulia.rabal@pharmacelera.com

Table of Content

Datasets used for retrospective validation 3

Citation S1. References for warheads collection 8

Citation S2. References for docking functions 8

References………………………………………………………………………………10

Appendix 1. Overview of the entire workflow, Evotec_Covalent_Processing_forGold..12

Appendix 2. Table of covalent warheads.. 13

Table of tables

Table S1. Details on the six study cases used in this study. 3

Table S2. The ROC-AUC values for the six targets and their corresponding protein structures obtained after GOLD covalent docking using different scoring functions. The best results for target proteins are indicated in bold (across the rows). 3

Table S3. The ROC-AUC values for the two targets and their corresponding protein structures obtained after GOLD non-covalent docking using different scoring functions. The best results for target proteins are indicated in bold (across the rows)...................... 4

Table S4. The Enrichment Factors, EF 1% and EF 5% values for the six targets and their corresponding protein structures obtained after GOLD covalent docking using different scoring functions. The best results for target proteins are indicated in bold (across the rows)……………………………………………………………………………...4

Table S5. The Enrichment Factors, EF 1% and EF 5% values for the two targets and their corresponding protein structures obtained after GOLD non-covalent docking using different scoring functions. The best results for target proteins are indicated in bold (across the rows)………………………………………………………………........4

Table of figures

Figure S1. A partial overview of the nodes that are dedicated to the standardization of the input molecules. 5

Figure S2. Workflow for the compound reaction. 5

Figure S3. Example of the transformation required by GOLD for covalent docking. a) With cysteine, b) with threonine 5

Figure S4. Example of reaction depending on the protein residue: a) cysteine, b) threonine…………………………………………………………………………….....6

Figure S5. The boxplots show the distribution of EF 1% values for the different scoring methods (GoldScore, ASP, ChemScore, PLP). For the six considered cases, ASP and PLP showed good performance by generating higher means as compared to GoldScore and ChemScore. 6

Figure S6. The boxplots show the distribution of EF 5% values for the different scoring methods (GoldScore, ASP, ChemScore, PLP). For the six considered cases, all scoring functions exhibited comparable performance in terms of EF5%....................................... 6

Figure S7. ROC curves for OTUB2. 8

Figure S7. ROC curves for EGFR (1XKK) 8

Datasets used for retrospective validation

Six study cases were compiled from the literature: NUDT7 (Scarpino *et al.*, 2021), OTUB2 (Scarpino *et al.*, 2021), EGFR, Cathepsin K, XPO1, and HCV NS3 protease (Toledo Warshaviak *et al.*, 2014). All information concerning the seven cases is listed in **Table S1**. The protein structures were downloaded from the Protein Data Bank (PDB) (Berman *et al.*, 2000; Burley *et al.*, 2017). The structures of the ligands of OTUB2 and NUDT7 were downloaded as SMILES from the supporting information (Scholz, C. *et al.,* 2015) The active ligands were constructed for EGFR, XPO1, Cathepsin K, and HCV NS3 protease from the supporting information (Toledo Warshaviak *et al.*, 2014). For these test cases, decoys with similar physicochemical properties and sharing the same warhead to the known actives were carefully chosen from a library of commercially available covalent compounds to achieve a reasonable number of inactives.

**Protein Target**	**Targeted Residue**	**Active compounds**	**Inactive Compounds**	**PDB ID**	**Reference**
NUDT7	Cys 73	29	587	5QHA	Scarpino *et al.*, 2021
OTUB2	Cys 51	41	558	5QIV	Scarpino *et al.*, 2021
EGFR	Cys 797	33	4046	2ITY, 1XKK	Toledo Warshaviak *et al.*, 2014
Cathepsin K	Cys 25	21	871	1YT7	Toledo Warshaviak *et al.*, 2014
XPO1	Cys 539	10	1968	4GMX	Toledo Warshaviak *et al.*, 2014
HCV NS3 protease	Ser 139	19	963	2F9U	Toledo Warshaviak *et al.*, 2014

Table S1. *Details on the six study cases used in this study.*

Target	PDB ID	GoldScore	ASP	ChemScore	PLP
NUDT7	5QHA	0.70	0.66	**0.77**	0.73
OTUB2	5QIV	**0.81**	0.77	0.73	0.71
EGFR	1XKK	0.84	**0.95**	0.86	0.82
	2ITY	0.91	**0.98**	0.94	0.90
Cathepsin K	1YT7	0.70	0.27	**0.83**	0.68
XPO1	4GMX	0.61	**0.67**	0.60	0.60
HCV NS3 protease	2F9U	0.57	0.42	0.55	**0.62**
Average AUC		0.73	0.67	**0.75**	0.72

Table S2. *The ROC-AUC values for the six targets and their corresponding protein structures obtained after GOLD covalent docking using different scoring functions. The best results for target proteins are indicated in bold (across the rows).*

Target	PDB ID	GoldScore	ASP	ChemScore	PLP
NUDT7	5QHA	0.68	**0.72**	0.62	0.69
OTUB2	5QIV	**0.85**	0.76	0.81	0.67

Table S3. *The ROC-AUC values for the two targets and their corresponding protein structures obtained after GOLD non-covalent docking using different scoring functions. The best results for target proteins are indicated in bold (across the rows).*

**Target**	**PDB ID**	**Enrichment factors**	**GoldScore**	**ASP**	**ChemScore**	**PLP**
NUDT7	5QHA	EF1%	3.45	0	3.45	**6.90**
		EF5%	0.69	1.38	2.07	**3.45**
OTUB2	5QIV	EF1%	0	**2.44**	**2.44**	**2.44**
		EF5%	**2.93**	2.44	**2.93**	1.95
EGFR	1XKK	EF1%	24.24	**39.39**	15.15	12.12
		EF5%	9.70	**12.73**	7.27	6.06
	2ITY	EF1%	21.21	**48.49**	18.18	33.33
		EF5%	12.12	**16.36**	12.12	10.91
Cathepsin K	1YT7	EF1%	4.76	0	**19.05**	14.29
		EF5%	3.81	0	**8.57**	6.67
XPO1	4GMX	EF1%	0	**30**	0	20
		EF5%	4	**8**	0	4
HCV NS3 protease	2F9U	EF1%	5.26	0	0	**10.53**
		EF5%	**4.21**	1.05	2.11	**4.21**

Table S4. *The Enrichment Factors, EF 1% and EF 5% values for the six targets and their corresponding protein structures obtained after GOLD covalent docking using different scoring functions. The best results for target proteins are indicated in bold (across the rows).*

**Target**	**PDB ID**	**Enrichment factors**	**GoldScore**	**ASP**	**ChemScore**	**PLP**
NUDT7	5QHA	EF1%	2.72	1.1	1.8	**4.8**
		EF5%	0.57	1.5	1.82	**2.97**
OTUB2	5QIV	EF1%	0.8	**2.44**	**2.44**	**2.44**
		EF5%	**1.65**	2.44	1.54	0.9

Table S5. *The Enrichment Factors, EF 1% and EF 5% values for the two targets and their corresponding protein structures obtained after GOLD non-covalent docking using different scoring functions. The best results for target proteins are indicated in bold (across the rows).*

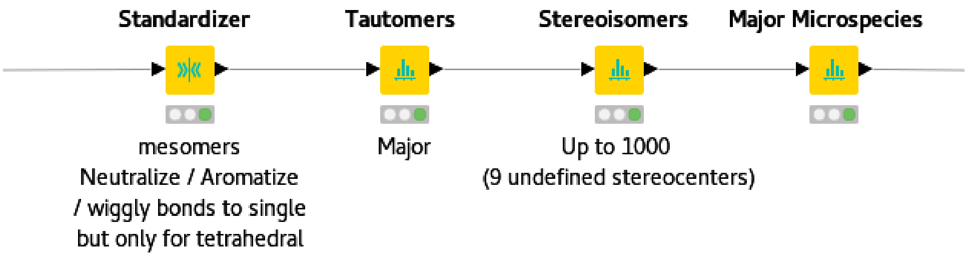

Figure S1. *A partial overview of the nodes that are dedicated to the standardization of the input molecules.*

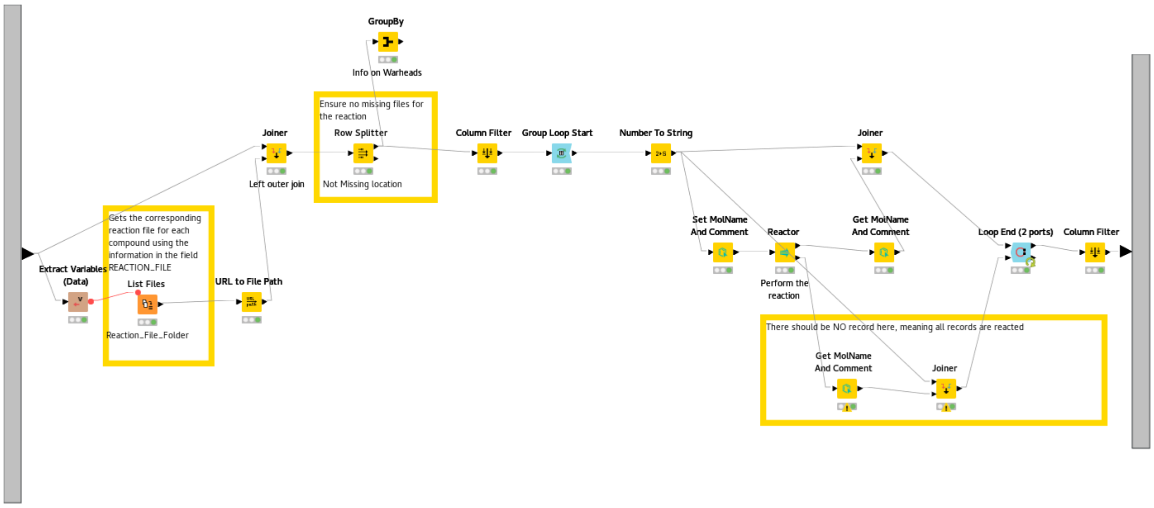

Figure S2. *Workflow for the compound reaction.*

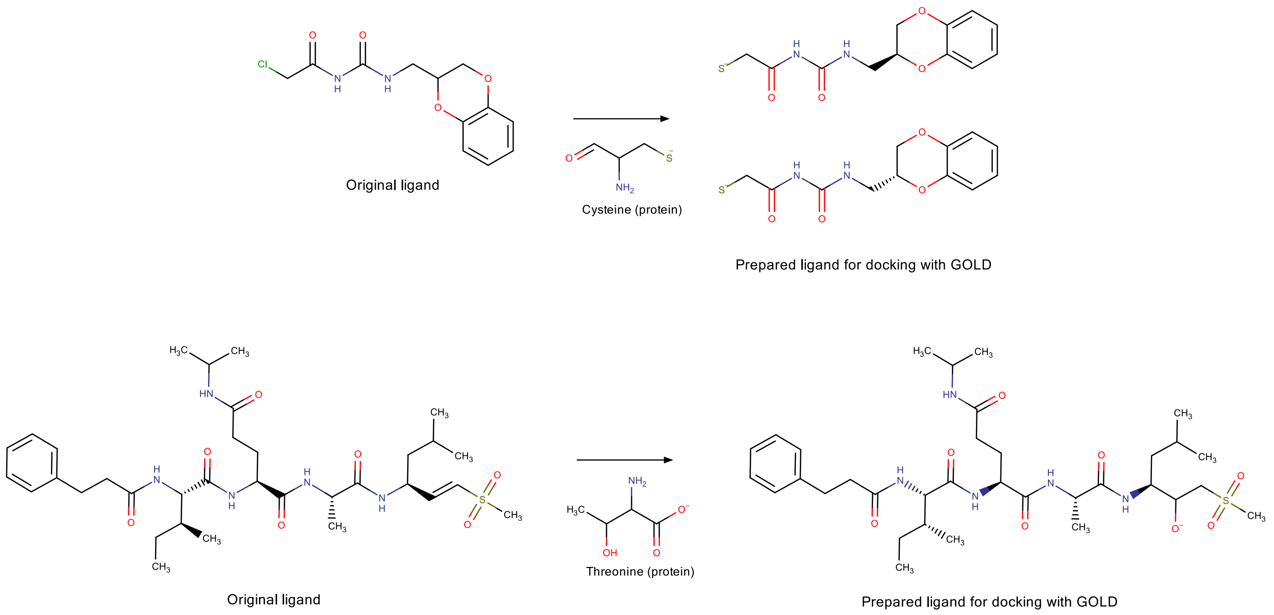

Figure S3. *Example of the transformation required by GOLD for covalent docking. a) With cysteine, b) with threonine*

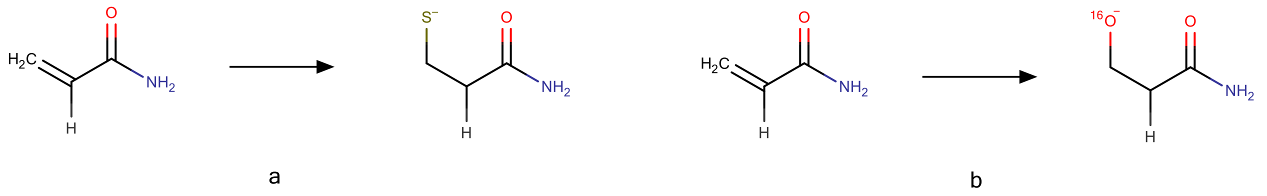

Figure S4. *Example of reaction depending on the protein residue: a) cysteine, b) threonine.*

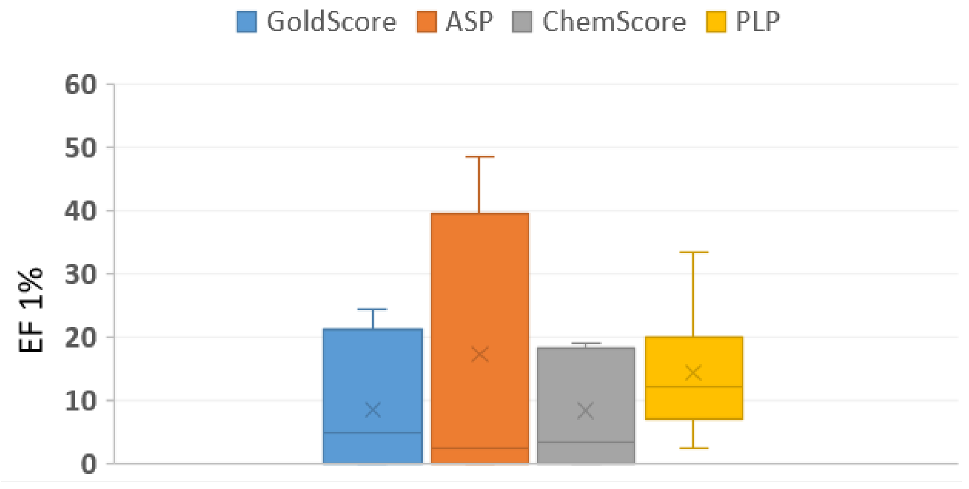

Figure S5*. The boxplots show the distribution of EF 1% values for the different scoring methods (GoldScore, ASP, ChemScore, PLP). For the six considered cases, ASP and PLP showed good performance by generating higher means as compared to GoldScore and ChemScore.*

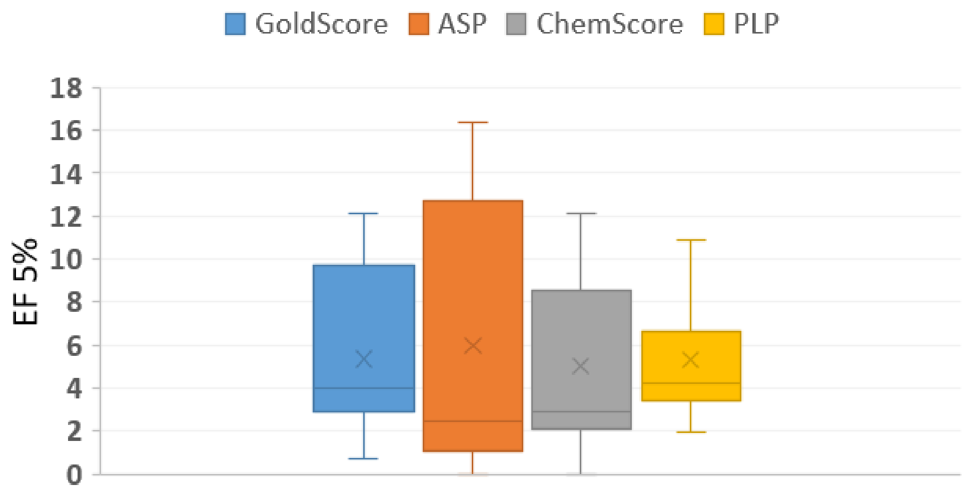

Figure S6. *The boxplots show the distribution of EF 5% values for the different scoring methods (GoldScore, ASP, ChemScore, PLP). For the six considered cases, all scoring functions exhibited comparable performance in terms of EF5%.*

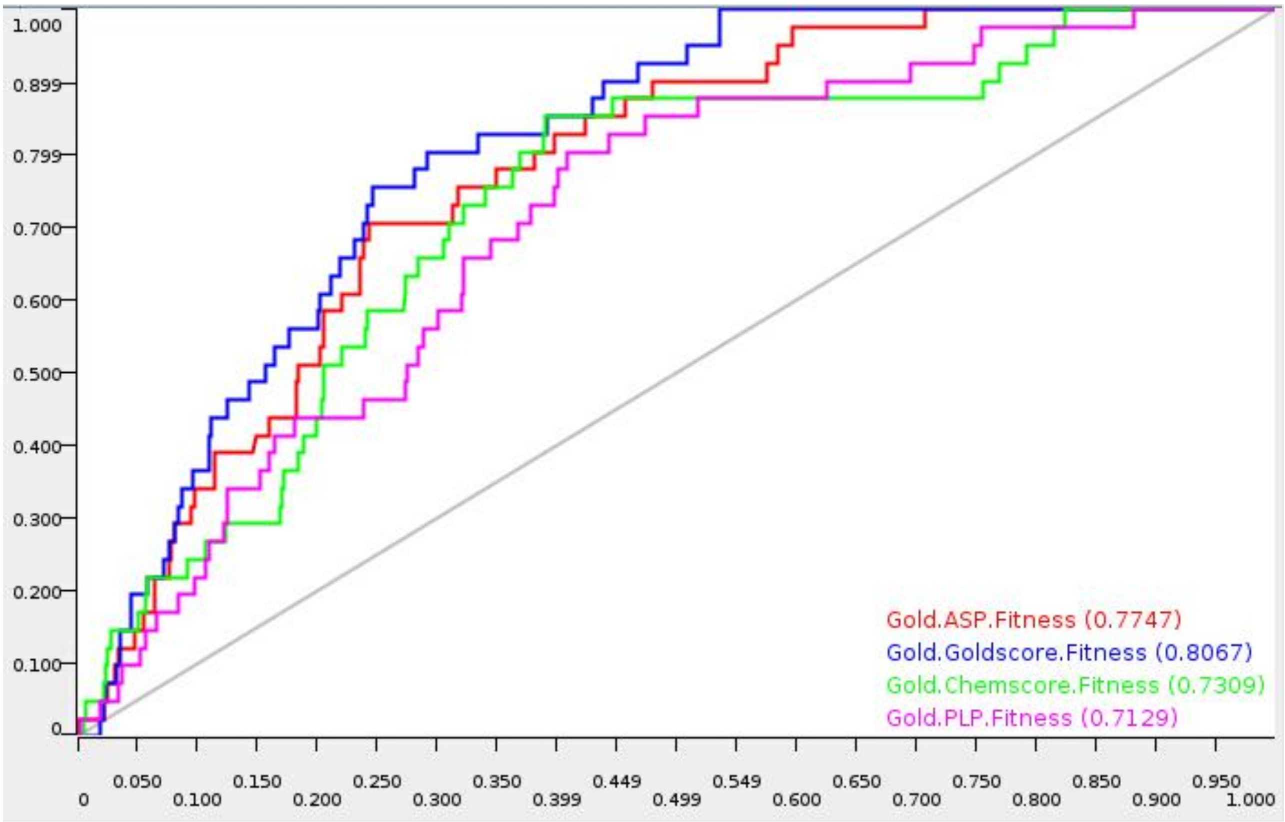

Figure S7. *ROC curves for OTUB2.*

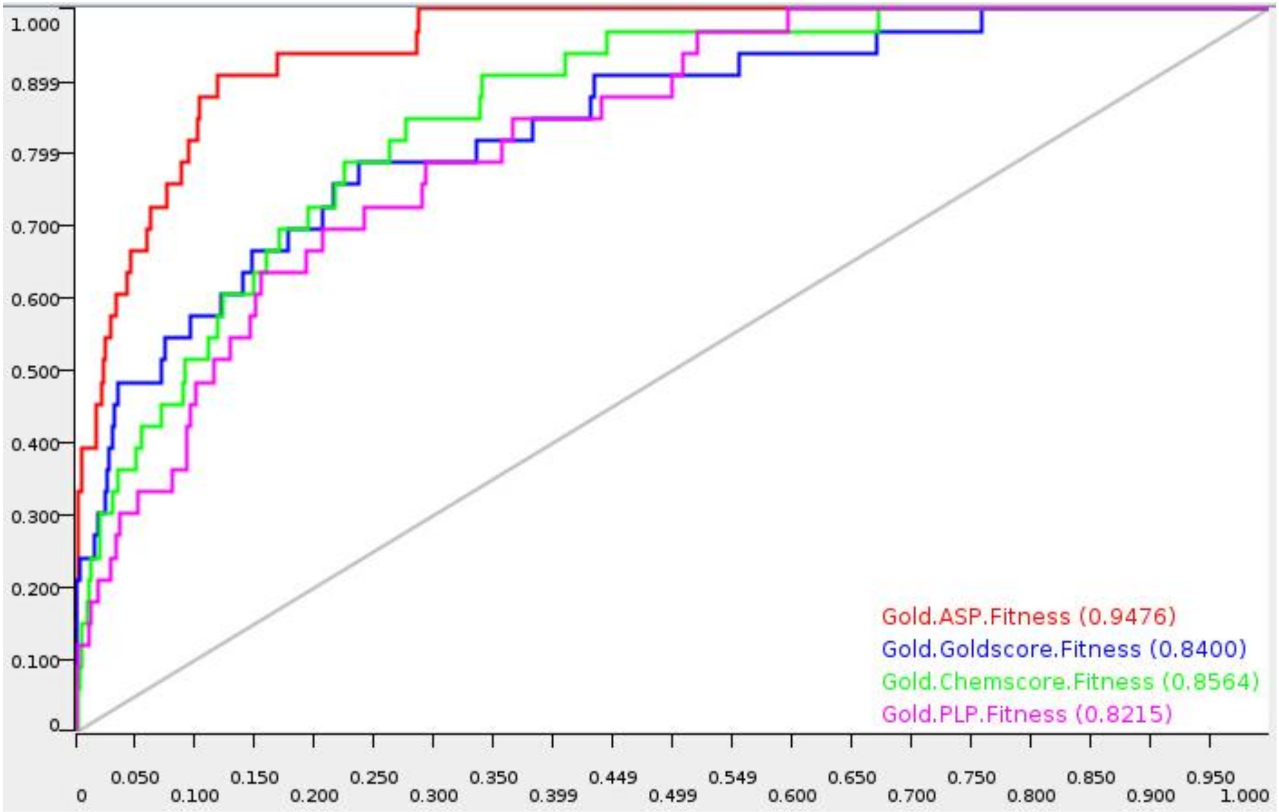

Figure S8. *ROC curves for EGFR (1XKK).*

Citation S1. References for warheads collection

Warheads were collected from the literature (Gehringer and Laufer, 2019; Bandyopadhyay and Gao, 2016; McAulay *et al.*, 2020; Tsou *et al.*, 2001; Ábrányi-Balogh *et al.*, 2018; Diaz and Yudin, 2017; Powers *et al.*, 2006; McGregor *et al.*, 2017; Scholz *et al.*, 2015; Shiu *et al.*, 2009; Klöck *et al.*, 2014; Dalton *et al.*, 2018; Jöst *et al.*, 2014; Xia *et al.*, 2014; Sutanto *et al.*, 2020; Breidenbach *et al.*, 2020; Deaton *et al.*, 2005; Nacht *et al.*, 2013; Crawford and Weerapana, 2016; Shannon and Weerapana, 2015; Koniev *et al.*, 2014; Hoffer *et al.*, 2019; Baggio *et al.*, 2019).

Citation S2. References for Docking Functions.

Main reference for GOLD docking functions: (Verdonk *et al.*, 2003)

<u>Detailed explanation for GoldScore:</u>

The prepared compound datasets were covalently docked to the respective target protein structures using 4 different GOLD scoring functions: GoldScore, PLP, ASP, and ChemScore. The scoring procedure of poses obtained from covalent docking using GoldScore is briefly described below:

In standard docking, the GoldScore fitness function consists of four components:

- protein-ligand hydrogen bond energy (external H-bond),
- protein-ligand van der Waals (vdW) energy (external vdW)
- ligand internal vdW energy (internal vdW)
- ligand torsional strain energy (internal torsion)

However, when docking covalently bound ligands, the fitness function also includes a covalent term S(cov) which is calculated by first assessing the extent of clash and torsional strain arising from the covalent linkage, and finally, the net energy contribution from the covalent linkage is calculated. Briefly, the procedure for calculating S(Cov) is:

- The clash term is reduced so that no clash is registered for 1-2 or 1-3 van der Waals contacts around the link atoms in the protein and ligand.
- Torsion terms are added for the rotatable parts of the linkage.
- A valence-angle bending term is added to the overall energy to penalize poor link geometries

S(Cov) term can be recognized in the docking output file as GoldScore. Covalent.Energy (Covalent bonding contribution to the GoldScore value). As we sampled 10 poses per ligand for the covalent ligand, it allowed us to explore the conformational space of the ligand to some extent (each pose is

different from the other by a minimum of 1.5 Å). The best poses can be differentiated from the inferior ones based on docking scores that consider both non-covalent interactions with the protein and the strength of the covalent bond between the warhead and the Cys or Ser residue of the protein. Only covalent linkages with proper torsion angles and minimum clash would likely allow the ligand to get accommodated favorably in the binding site.

References

Ábrányi-Balogh,P. *et al.* (2018) A road map for prioritizing warheads for cysteine targeting covalent inhibitors. *Eur. J. Med. Chem.*, **160**, 94–107.

Baggio,C. *et al.* (2019) Aryl-fluorosulfate-based Lysine Covalent Pan-Inhibitors of Apoptosis Protein (IAP) Antagonists with Cellular Efficacy. *J. Med. Chem.*, **62**, 9188–9200.

Bandyopadhyay,A. and Gao,J. (2016) Targeting biomolecules with reversible covalent chemistry. *Curr. Opin. Chem. Biol.*, **34**, 110–116.

Berman,H.M. *et al.* (2000) The Protein Data Bank. *Nucleic Acids Res.*, **28**, 235–42.

Breidenbach,J. *et al.* (2020) Coumarin as a structural component of substrates and probes for serine and cysteine proteases. *Biochim. Biophys. Acta - Proteins Proteomics*, **1868**, 140445.

Burley,S.K. *et al.* (2017) Protein Data Bank (PDB): The Single Global Macromolecular Structure Archive. *Methods Mol. Biol.*, **1607**, 627–641.

Crawford,L.A. and Weerapana,E. (2016) A tyrosine-reactive irreversible inhibitor for glutathione S-transferase Pi (GSTP1). *Mol. Biosyst.*, **12**, 1768–1771.

Dalton,S.E. *et al.* (2018) Selectively Targeting the Kinome-Conserved Lysine of PI3Kδ as a General Approach to Covalent Kinase Inhibition. *J. Am. Chem. Soc.*, **140**, 932–939.

Deaton,D.N. *et al.* (2005) Novel and potent cyclic cyanamide-based cathepsin K inhibitors. *Bioorg. Med. Chem. Lett.*, **15**, 1815–1819.

Diaz,D.B. and Yudin,A.K. (2017) The versatility of boron in biological target engagement. *Nat. Chem.*, **9**, 731–742.

Gehringer,M. and Laufer,S.A. (2019) Emerging and Re-Emerging Warheads for Targeted Covalent Inhibitors: Applications in Medicinal Chemistry and Chemical Biology. *J. Med. Chem.*, **62**, 5673–5724.

Hoffer,L. *et al.* (2019) CovaDOTS: In Silico Chemistry-Driven Tool to Design Covalent Inhibitors Using a Linking Strategy. *J. Chem. Inf. Model.*, **59**, 1472–1485.

Jöst,C. *et al.* (2014) Promiscuity and Selectivity in Covalent Enzyme Inhibition: A Systematic Study of Electrophilic Fragments. *J. Med. Chem.*, **57**, 7590–7599.

Klöck,C. *et al.* (2014) Discovery of Potent and Specific Dihydroisoxazole Inhibitors of Human Transglutaminase 2. *J. Med. Chem.*, **57**, 9042–9064.

Koniev,O. *et al.* (2014) Selective Irreversible Chemical Tagging of Cysteine with 3-Arylpropiolonitriles. *Bioconjug. Chem.*, **25**, 202–206.

McAulay,K. *et al.* (2020) Alkynyl Benzoxazines and Dihydroquinazolines as Cysteine Targeting Covalent Warheads and Their Application in Identification of Selective Irreversible Kinase Inhibitors. *J. Am. Chem. Soc.*, **142**, 10358–10372.

McGregor,L.M. *et al.* (2017) Expanding the Scope of Electrophiles Capable of Targeting K-Ras Oncogenes. *Biochemistry*, **56**, 3178–3183.

Nacht,M. *et al.* (2013) Discovery of a Potent and Isoform-Selective Targeted Covalent Inhibitor of the Lipid Kinase PI3Kα. *J. Med. Chem.*, **56**, 712–721.

Powers,J.P. *et al.* (2006) SAR and Mode of Action of Novel Non-Nucleoside Inhibitors of Hepatitis C NS5b RNA Polymerase. *J. Med. Chem.*, **49**, 1034–1046.

Scarpino,A. *et al.* (2021) WIDOCK: a reactive docking protocol for virtual screening of covalent inhibitors. *J. Comput. Aided. Mol. Des.*, **35**, 223–244.

Scholz,C. *et al.* (2015) DOCKTITE—A Highly Versatile Step-by-Step Workflow for Covalent Docking and Virtual Screening in the Molecular Operating Environment. *J. Chem. Inf. Model.*, **55**, 398–406.

Shannon,D.A. and Weerapana,E. (2015) Covalent protein modification: the current landscape of residue-specific electrophiles. *Curr. Opin. Chem. Biol.*, **24**, 18–26.

Shiu,H.-Y. *et al.* (2009) Electron-Deficient Alkynes as Cleavable Reagents for the Modification of Cysteine-Containing Peptides in Aqueous Medium. *Chem. - A Eur. J.*, **15**, 3839–3850.

Sutanto,F. *et al.* (2020) Covalent inhibitors: a rational approach to drug discovery. *RSC Med. Chem.*, **11**, 876–884.

Toledo Warshaviak,D. *et al.* (2014) Structure-based virtual screening approach for discovery of covalently bound ligands. *J. Chem. Inf. Model.*, **54**, 1941–50.

Tsou,H.-R. *et al.* (2001) 6-Substituted-4-(3-bromophenylamino)quinazolines as Putative Irreversible Inhibitors of the Epidermal Growth Factor Receptor (EGFR) and Human Epidermal Growth Factor Receptor (HER-2) Tyrosine Kinases with Enhanced Antitumor Activity. *J. Med. Chem.*, **44**, 2719–2734.

Verdonk,M.L. *et al.* (2003) Improved protein-ligand docking using GOLD. *Proteins Struct. Funct. Bioinforma.*, **52**, 609–623.

Xia,G. *et al.* (2014) A Chemical Tuned Strategy to Develop Novel Irreversible EGFR-TK Inhibitors with Improved Safety and Pharmacokinetic Profiles. *J. Med. Chem.*, **57**, 9889–9900.

Appendix 1. Overview of the entire workflow: Evotec_Covalent_Processing_forGold

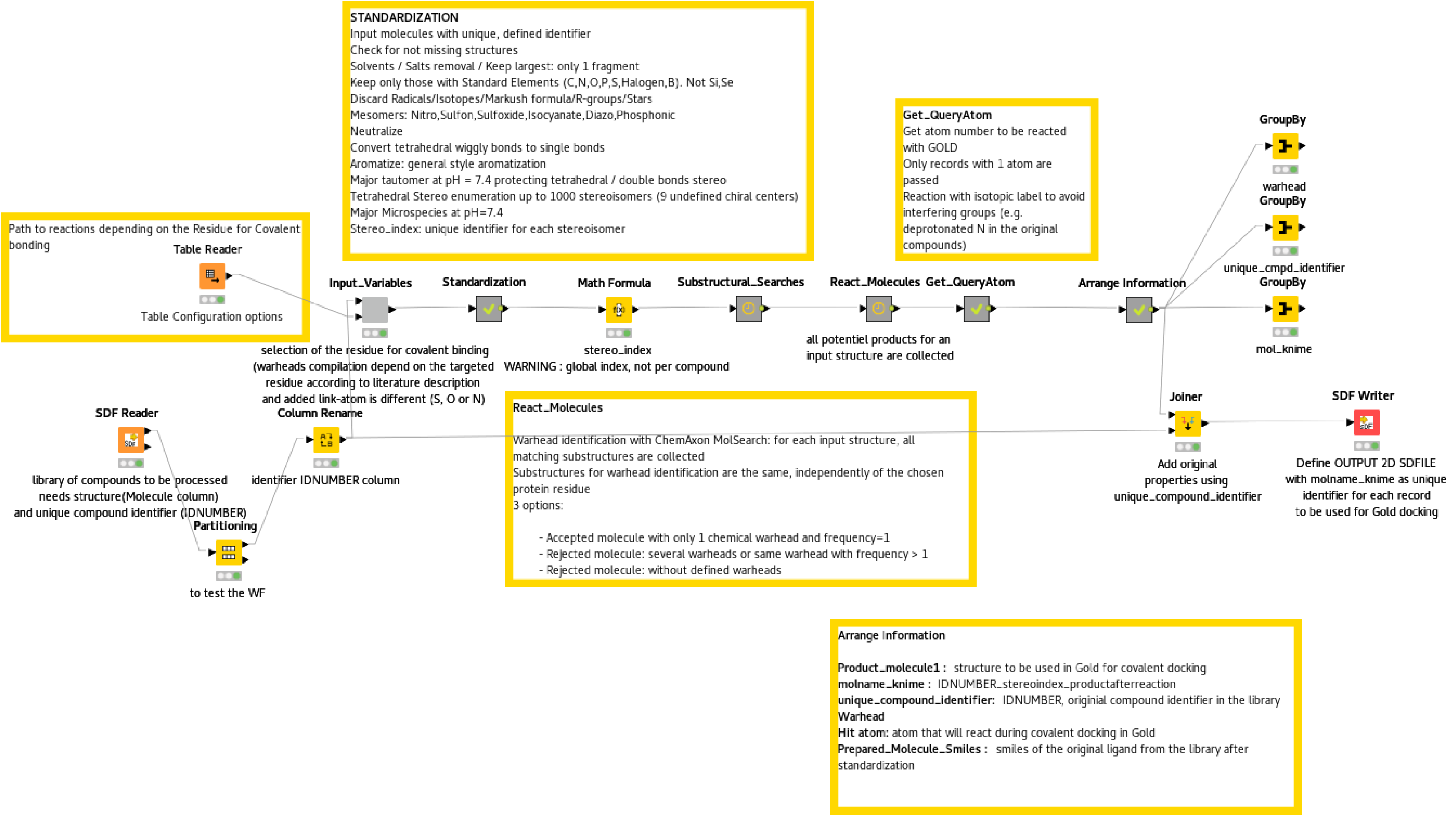

Appendix 2. Table of covalent warheads.

The structures provided can include SMARTS features (e.g. propiolonitrile). A detailed explanation of different notations can be found here: https://docs.chemaxon.com/display/docs/smarts.md.

Structure	Warhead Name	Protein residue
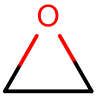	Epoxide	Cys, Lys, Ser, Thr, His
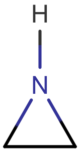	Aziridine	Cys, Asp, Glu
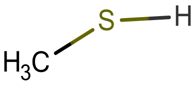	Thiol	Cys
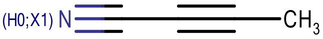	Propiolonitrile	Cys
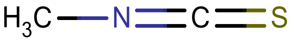	Isothiocyanates	Cys, Lys
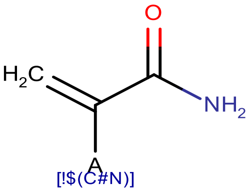	Acrylamide_AlphaSubstituted	Cys, Lys, Ser, Thr, His
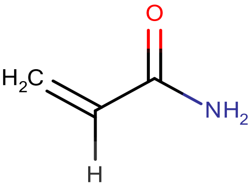	Acrylamide_AlphaH	Cys, Lys, Ser, Thr, His
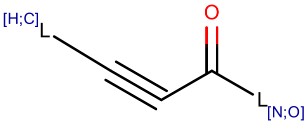	Propiolamide	Cys
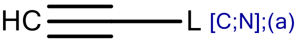	Alkynyl_Heteroarene	Cys
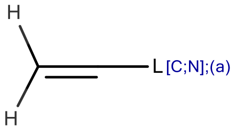	Vinyl_Heteroarene	Cys

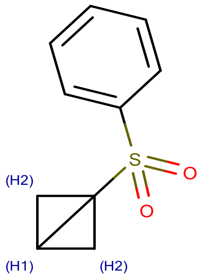	Arylsulfonyl_Bicyclobutane	Cys
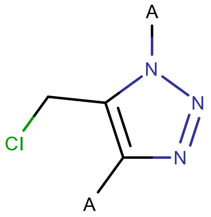	Chloromethyl_triazole	Cys
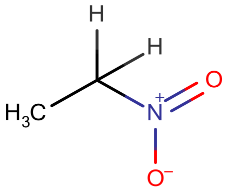	Nitroalkane	Cys
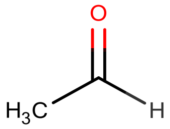	Aldehyde	Cys, Ser, Thr
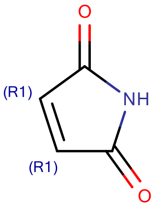	Maleimide	Cys
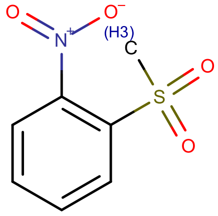	methylsulfonyl_nitrobenzoates	Cys
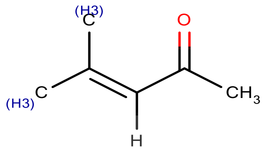	Dimethyl_Vinyl_Ketone	Cys, Lys
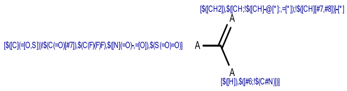	Michael_alkene_acceptor	Cys, Lys, Ser, Thr, His
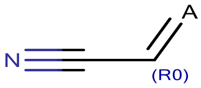	acrylonitrile	Cys, Lys, His

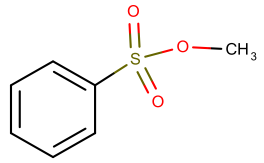	sulfonate_leaving_group	Cys, Asp, Glu, Tyr
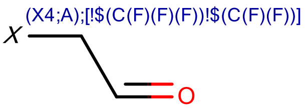	Alpha halo carbonyl	Cys
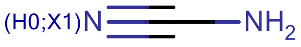	Cyanamide_all	Cys, Lys, Ser, Thr
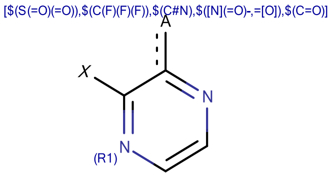	2halo-3EWG-pyrazine	Cys
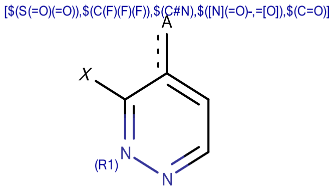	2halo-3EWG-pyridazine	Cys
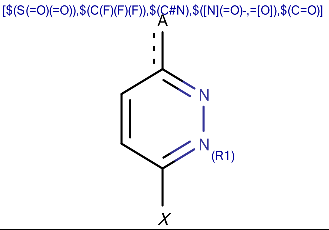	2halo-5EWG-pyridazine	Cys
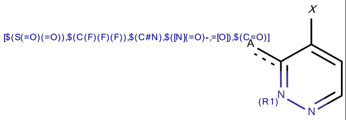	3halo-2EWG-pyridazine	Cys
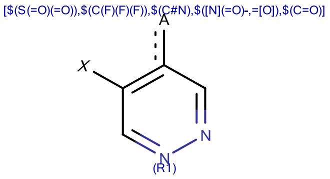	3halo-4EWG-pyridazine	Cys
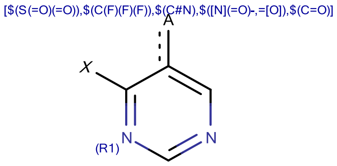	4halo-5EWG-pyrimidine	Cys
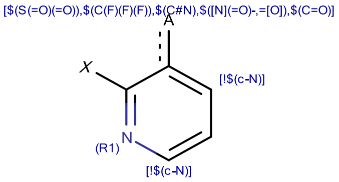	2halo-3EWG-pyridine	Cys
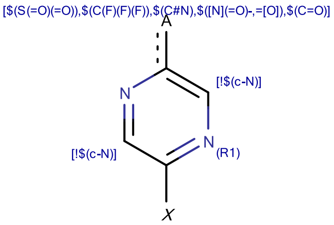	2halo-5EWG-pyrazine	Cys

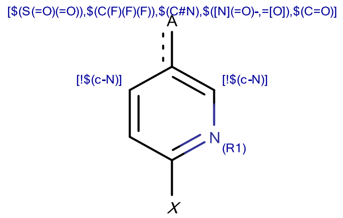	2halo-5EWG-pyridine	Cys
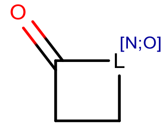	beta_lactam_lacton	Cys, Ser, Thr
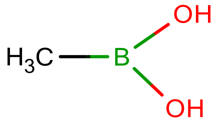	boronate	Ser, Thr
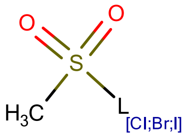	sulfonylhalide_notF	Cys, Lys
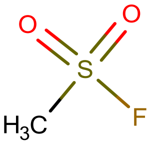	sulfonylfluoride	Lys, Ser, Thr, His, Tyr
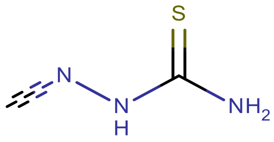	Thiosemicarbazone	Cys
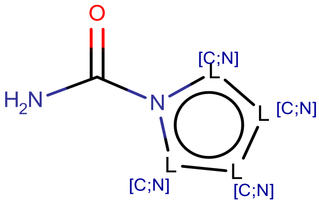	azole_urea	Cys, Ser
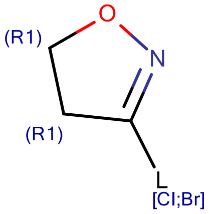	bromo_chloro_dihydroisoxazole	Cys
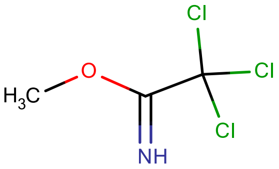	trichloroethanimidate	Cys
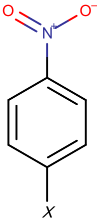	4-halonitrophenyl	Cys

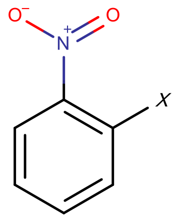	2-halonitrophenyl	Cys
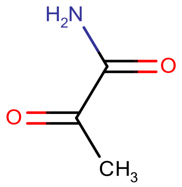	Alpha_Ketoamide	Cys, Ser, Thr
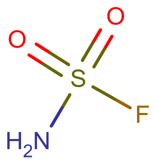	sulfamoyl_fluoride	Lys, Ser, Thr, Tyr
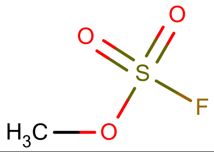	fluorosulfate	Lys, Ser, Thr, Tyr
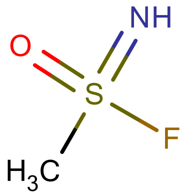	sulfonimidoyl_fluoride	Lys, Tyr
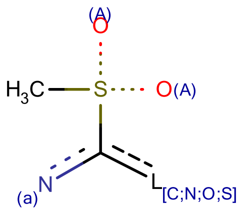	Alpha_sulphonyl N-aromatic	Cys
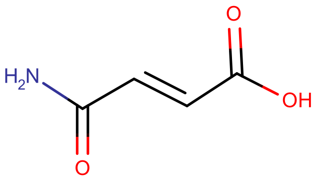	fumarate_amide	Cys
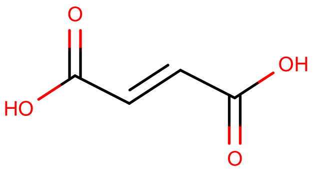	fumarate	Cys
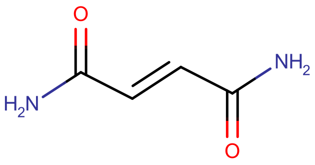	Butendiamide	Cys
(H1)C$\equiv$CH	Terminal_Alkyne	Cys

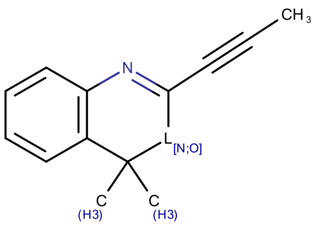	Alkynyl_benzoxazine	Cys
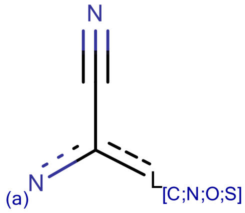	Alpha_nitrile_N-aromatic	Cys, Ser, Thr
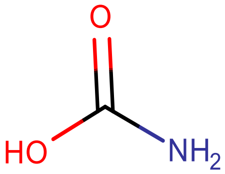	Carbamate	Ser
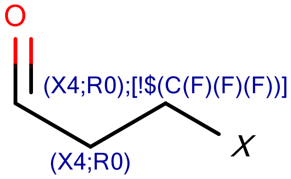	Beta_halo_carbonyl	Cys
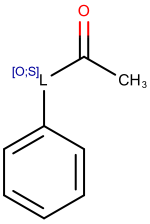	Activated_ester	Lys, Ser
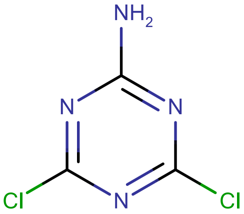	Dichloro_triazine	Lys, Tyr
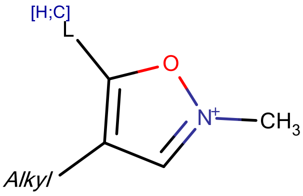	N-methyl_isoxazolium	Cys, Asp, Glu
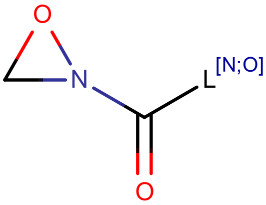	Oxaziridine	Met

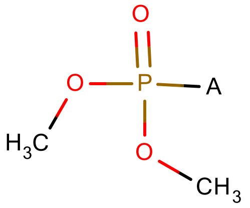	Phophonate	Ser
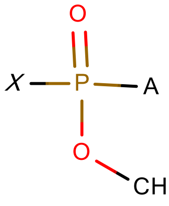	Phosphonylhalide	Ser
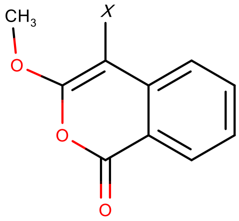	Isocoumarin	Cys, Ser
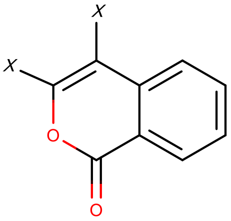	Dihalo_Isocoumarin	Cys, Ser
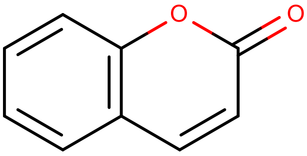	Coumarin	Cys, Ser
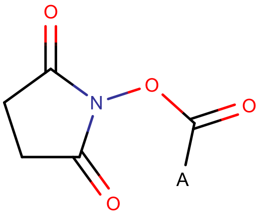	Succinimidyl_ester	Lys
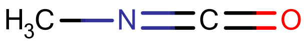	Isocyanate	Lys
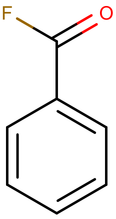	Benzoyl_Fluoride	Lys
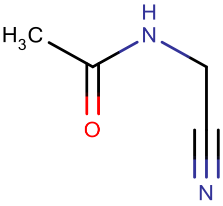	Amidoacetonitrile	Cys, Ser, Thr

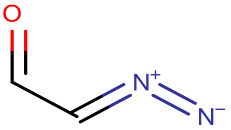	Diazomethyl_ketone	Cys
