# Supplementary material for: A toolkit for covalent docking with GOLD: from automated ligand preparation with KNIME to bound protein–ligand complexes
Source: Bioinform Adv. 2022 Nov 29;2(1):vbac090. doi: 10.1093/bioadv/vbac090 (PMC9722222; doi:10.1093/bioadv/vbac090)
Supplement: vbac090_Supplementary_Data [file vbac090_supplementary_data.zip › covalent_docking_helper_program_user_guide.pdf]

How to use `covalent_docking_helper.py`

About the program

The `covalent_docking_helper.py` is a ready-to-use program for performing covalent docking using GOLD. The program can be downloaded from the following url:

https://gitlab.com/seb-buch/covalent_docking_helper

Getting started

In the terminal, use the command change directory (cd) and go to the directory where the file `covalent_docking_helper.py` is located. To list the different parameters available run the commands:

```
module load csds
```

```
python covalent_docking_helper.py -h
```

Prerequisites for running covalent docking

The following files are needed:

1. A ligand database provided as a .sdf file that contains a filed named 'Hit atom' to specify the atom number of the ligand's atom bound to the protein.
2. A GOLD configuration that is consistent with "atom-based" (as opposed to "substructure-based") covalent docking

Prepare the files to run the docking

The 3D conformations from the 2D file (obtained from the knime workflow) were generated using Corina. However, other programs can be used to prepare 3D conformations, if the user does not have access to Corina. The following commands were used:

```
module load corina
```

```
corina -d wh,errorfile=lig_prep_error.sdf,axchir,r2d,stergen,msc=2,names,preserve -o
pascom,lname,keepnames -i t=sdf -o t=sdf ligand_2D.sdf Ligand_3D.sdf
```

Run `covalent_docking_helper.py` with the sub command `prepare`. You can specify the GOLD configuration file with the option `--conf` (or `-c`). If not specified, the script will search for a `gold.conf` file. You may also specify the name of the output script that will be written for being submitted to SGE with the option `--output-job` (or `-o`). If not specifid, a job script named `run_my_covalent_docking.sh` will be created. Here is an example:

```
python covalent_docking_helper.py prepare --conf gold.conf -o job.sh
```

Note: In addition to `job.sh`, the script will also create many `gold_XXXXX.conf` (where XXXXX is number up to 99999) and as many `input_XXXXX.sdf` (provided the ligand database is `input.sdf`).

On exit, the script will specify the command to be used to submit the docking to the cluster. The command will looklike this one:

```
qsub –q cpu -t 1-120 -wd $(pwd) -o gold.log -tc 50 -j y job.sh
```

-t: to define the number of files

-tc: to define the number of concurrent docking jobs

-**q cpu** : IMPORTANT! to define the cpu queue in order to use the cpu nodes

Note: To ease the footprint on the cluster, this command will run no more than 50 concurrent docking jobs (`-tc` option).

Process results from covalent docking

When the docking jobs are completed successfully, run `covalent_docking_helper.py` with the sub command `process`.

You need to specify the original GOLD configuration file with the option `--conf` (or `-c`). If not specified, thescript will search for a `gold.conf` file. You may also specify the name of the two output files:

1. `--output-complexes` (or `-o`) to specify where to save the covalent complexes (docked ligand+protein). Default file is `complexes.mol2`.

2. `--output-poses` (or `-p`) to specify where to save the concatenated docked poses with the docking results (ligand only, no protein there). Default file is `poses.sdf`.

Here is an example:

```
python covalent_docking_helper.py process --conf gold.conf -o
complexes.mol2 -p poses.sdf
```

Merge the created database with MOE

1. Open `poses.sdf` with MOE and create a database named `poses.mdb`.
2. Create a database named `complexes.mdb` and import `complexes.mol2` in it and rename the field `mol` to `complex`.
3. Create a new field in `complexes.mdb` named `complex_id` that contain the extracted name for the field `complex`. (right click on the `complex` field, then `Name`>`Extract...`)
4. Merge `poses.mdb` and `complexes.mdb` into `docking.mdb` using MOE's Database Viewer: `File` > `Merge...`. Use the field named `complex_id` (present in both databases) as the key to identify related entries. There should be no duplicates entries nor ones that are not present in both databases.
5. Simply open `docking.mdb` to browse the docking results that contains everything!
